# Cigarette smoke–induced induction of antioxidant enzyme activities in airway leukocytes is absent in active smokers with COPD

**DOI:** 10.3402/ecrj.v2.27837

**Published:** 2015-07-16

**Authors:** Rosamund E. Dove, Pheneatia Leong-Smith, Ester Roos-Engstrand, Jamshid Pourazar, Mittal Shah, Annelie F. Behndig, Ian S. Mudway, Anders Blomberg

**Affiliations:** 1 Department of Public Health and Clinical Medicine, Division of Medicine/Respiratory Medicine and Allergy, Umeå University, Umeå, Sweden; 2 MRC-PHE Centre for Environment and Health, School of Biomedical Sciences, Kings College London, London, UK

**Keywords:** oxidative stress, smoking, bronchoscopy, bronchoalveolar lavage

## Abstract

**Background:**

Oxidative injury to the airway has been proposed as an important underlying mechanism in the pathogenesis of chronic obstructive pulmonary disease (COPD). As the extent of oxidant-mediated damage is dependent on the endogenous antioxidant defences within the airways, we examined whether COPD was associated with deficiencies in the antioxidant network within the respiratory tract lining fluids (RTLFs) and resident airway leukocytes. We hypothesised that COPD would be associated with both basal depression of antioxidant defences and impaired adaptive antioxidant responses to cigarette smoke.

**Methods:**

Low molecular weight and enzymatic antioxidants together with metal-handling proteins were quantified in bronchoalveolar lavage fluid and airway leukocytes, derived from current (*n*=9) and ex-smoking COPD patients (*n*=15), as well as from smokers with normal lung function (*n*=16) and healthy never smokers (*n*=13).

**Results:**

Current cigarette smoking was associated with an increase in ascorbate and glutathione within peripheral RTLFs in both smokers with normal lung function compared with healthy never smokers and in COPD smokers compared with COPD ex-smokers. In contrast, intra-cellular antioxidant enzyme activities (glutathione peroxidase, glutathione reductase, and catalase) were only up-regulated in smokers with normal lung function compared with healthy never smokers and not in actively smoking COPD patients relative to COPD ex-smokers.

**Conclusions:**

We found no evidence of impaired basal antioxidant defences, within either the RTLFs or airway leukocytes in stable ex-smoking COPD patients compared with healthy never smoking controls. Current cigarette smoking induced an up-regulation of low molecular weight antioxidants in the RTLFs of both control subjects with normal lung function and patients with COPD. Importantly, the present data demonstrated a cigarette smoke–induced increase in intra-cellular antioxidant enzyme activities only within the smokers with normal lung function, implying that patients with COPD who continue to smoke will experience enhanced oxidative stress, prompting disease progression.

Oxidative stress, together with inflammation and protease-antiprotease imbalance in the airways, represents the classic pathologic triad associated with chronic obstructive pulmonary disease (COPD) (1). The involvement of oxidative stress, related to chronic inflammation and periods of acute inflammation during exacerbation, is supported by evidence of increased oxidative damage marker concentrations within the respiratory tract lining fluids (RTLFs) of patients with COPD, assessed using induced sputum (2–4), exhaled gases (5, 6), and exhaled breath condensate (EBC) (7–9). Evidence of increased signalling through redox sensitive pathways (10, 11) and oxidative damage within the respiratory epithelium (12, 13) has also been reported in COPD patients and related to disease severity.

Under normal circumstances, the airway is particularly rich in antioxidant species, both within the airway epithelium and in the RTLF. Small molecular weight compounds and families of antioxidant enzymes act in synergy to scavenge reactive oxygen species (ROS) to maintain a ‘reducing’ intra- and extra-cellular environment at the air–lung interface. In addition, other processes act indirectly to maintain redox homeostasis, including chelator proteins that sequester redox catalysts such as iron or copper. To date, there is little information on the antioxidant defences within the lung in COPD beyond a single study examining low molecular weight antioxidant concentrations in lavage and saliva samples (14), and evidence of reduced antioxidant enzyme mRNA expression (haeme oxygenase-1, glutathione peroxidase (GPx) 2 and NAD(P)H:quinone oxidoreductase 1) in alveolar macrophages from patients with emphysema (15). Much of our current understanding of antioxidant defences in COPD has been restricted to circulating lymphocytes, where evidence of decreased antioxidant concentrations and enzyme activities has been reported (16).

In the present study, we hypothesised that COPD would be associated with impaired antioxidant defences at the air–lung interface, both within the RTLF and the resident airway leukocytes, relative to both healthy and smoking history–matched controls. We further hypothesised that the extent of oxidative stress in the RTLF would be related to disease severity, the presence and extent of airway inflammation and measured lung function. Bronchoalveolar lavage (BAL) was employed to sample the peripheral airways of COPD patients (ex-smokers and current smokers), smokers with normal lung function and healthy never smokers. A panel of low molecular weight antioxidants and metal chelator proteins was quantified in the cell-free lavage, and antioxidant enzyme activities were quantified in BAL leukocytes.

## Methods

### Subject demographics

In total, 56 subjects were recruited: 13 healthy never smokers (age 57–74 years) with normal lung function [forced expiratory volume in 1 second (FEV_1_) % predicted>80%]; 16 smokers (age 50–71 years) with a smoking history of more than 10 pack-years and with normal lung function; 16 ex-smoking COPD patients with smoking cessation of more than 5 years prior to inclusion (age 53–77 years) and 11 COPD patients (age 55–75 years) who had continued to smoke following their COPD diagnosis. Both men and women were included. Subject demographics are given in Table 1. All participants in the study were non-atopic and without any respiratory tract infection within a 6-week period prior to the study. All subjects underwent a detailed medical consultation, including ECG and chest X-ray prior to inclusion. COPD patients had moderate-to-severe disease, Stage II–III, according to the GOLD criteria [FEV_1_ 30–80% of predicted, FEV_1_/VC (vital capacity) less than 0.7], with a smoking history of at least 10 pack-years and no evidence of other concomitant disease. Patients were required to have no history of an exacerbation during a period of at least 3 months prior to inclusion. The only medication permitted during the study was short-acting β2-agonists and/or anti-cholinergic drugs. Prior to study inclusion, long-acting bronchodilators and inhaled corticosteroids were not allowed within 2 and 4 weeks, respectively. All COPD subjects were clinically stable. All participants provided informed consent, and the local Ethical Review Board of Umeå University approved the study that was performed in accordance with the declaration of Helsinki. Subjects were recruited at the Department of Public Health and Clinical Medicine, Division of Medicine/Respiratory Medicine at Umeå University, and all clinical investigations, including bronchoscopy, were carried out at the University Hospital, Umeå, Sweden.

**Table 1:** Subject demographics for healthy never smokers, smokers with normal lung function (LF), COPD ex-smokers, and COPD current smokers

	Healthy never smokers (*n*=13)	Smokers with normal LF (*n*=16)	COPD ex-smokers (*n*=16)	COPD smokers (*n*=11)	KW test
Age (mean+range)	67 (57–74)	61 (50–71)*a*	68 (53–77)	64 (55–75)	*P*=0.014
Gender (m/f)	7/6	7/9	11/5	3/8	
Pack-years (mean+range)	0	37 (18–95)*a*	34 (5–68)*c*	35 (13–80)	*P*<0.001
FVC (median, IQR, L)	4.1 (3.6–4.7)	4.0 (3.1–4.9)	2.9 (2.3–3.0)*c*	2.4 (2.2–2.6)*b*	*P*<0.001
FVC% of predicted (%)	104.0 (95.0–116.0)	114.5 (107.0–122.3)	73.0 (66.0–77.5)*c*	85.0 (67.5–91.0)*b*	*P*<0.001
FEV_1_ (median, IQR, L)	3.0 (2.2–3.4)	3.2 (2.6–3.8)	1.4 (1.0–1.9)*c*	1.3 (1.1–1.6)b	*P*<0.001
FEV_1_% of predicted (%)	100.0 (91.0–118.0)	113.0 (104.0–115.8)	51.0 (42.0–64.0)*c*	60.0 (39.0–69.0)*b*	*P*<0.001
Reversibility (%)	1.0 (−0.5–4.4)	2.7 (1.2–4.9)	17.1 (12.0–24.4)*c*	6.2 (1.9–15.1)*d*	*P*<0.001
TLC (median, IQR, L)	nd	6.8 (5.4–8.1)	7.0 (6.1–7.6)	6.0 (5.3–6.5)	NS
IC (median, IQR, L)	nd	3.1 (2.7–3.6)	2.6 (2.2–3.0)	2.0 (1.7–2.5)b	*P*=0.006
MEF_50_ (median, IQR, L s^−1^)	nd	2.76 (2.15–3.64)	0.37 (0.28–0.70)	0.72 (0.40–0.79)*b*	*P*<0.001
DL_CO_^SB^ (median, IQR, mmol min^−1^ kPa^−1^)	nd	7.4 (6.1–7.8)	5.7 (3.9–6.3)	5.2 (4.8–5.7)*b*	*P*<0.001
BAL-recovery (%)	50.0 (44.0–56.0)	53.0 (48.8–63.8)	36.0 (29.0–48.0)*c*	39.0 (29.0–48.0)	*P*=0.025

All data are expressed as medians with either the inter-quartile or full range, as indicated. Significant differences across groups were assumed at the 5% level using the Kruskal–Wallis one-way analysis of variance by ranks (significance illustrated in the far right hand column), with post-hoc testing between specified groups performed using the Mann–Whitney U Test.

Comparisons of individual groups were restricted to smokers with normal lung function (LF) versus healthy never smokers (*a*, *P*<0.05), smokers with normal LF versus COPD current smokers (*b*, *P*<0.05), healthy never smokers versus COPD ex-smokers (*c*, *P*<0.05), and COPD current versus COPD ex-smokers (*d*, *P*<0.05).

Lung function (LF) measurements are based on post-bronchodilator values, with evidence of % airway reversibility indicated. The European Coal and Steel Community (ECSC) reference values were employed for the lung function variables. Pack-years were calculated by multiplying the number of packs of cigarettes smoked per day by the number of years the person had smoked.

FVC=forced vital capacity; FEV_1_=forced expiratory volume in 1 second; TLC=total lung capacity; IC=inspiratory capacity; MEF_50_=maximum expiratory flow when 50% of the FVC has been exhaled; DL_CO_^SB^, lung diffusing capacity to carbon monoxide in a single breath; % BAL-recovery, based on a total instilled saline volume of 180 mLs; nd=not determined.

### Pulmonary function

Dynamic spirometry variables [VC, FVC (forced vital capacity), and FEV_1_] were measured pre- and 20 min post-bronchodilatation with 1 mg of terbutaline (Bricanyl^®^, Turbuhaler^®^; AstraZeneca, Södertälje, Sweden), using a Vitalograph spirometer (Buckingham, UK) and reversibility calculated. At least three satisfactorily performed and well-coordinated measurements of each variable were carried out, according to the recommendations of the American Thoracic Society (17). The diffusion capacity of carbon monoxide (DL_CO_) was obtained by the single-breath procedure.

### Sample collection and preparation

All subjects underwent bronchoscopy with bronchial wash (BW, 2×20 ml) to sample proximal airways and BAL (3×60 ml) to sample the more peripheral alveolar region using sterile sodium chloride (0.9%). Total cells and differential cell counts were determined in samples from both BW and BAL fluid, whereas determination of antioxidant concentrations and activities were restricted to the BAL fluid samples due to sample availability. Full details of the sampling protocol are outlined in the online supplement.

### Determination of antioxidant concentrations and activities

BAL fluid glutathione (GSH) concentrations were established using the glutathione disulphide (GSSG)-reductase-dithiobis-2-nitrobenzoic acid (DTNB) recycling method (18). BAL fluid ascorbate (AA) and urate (UA) were measured by reverse phase HPLC with electrochemical detection (19) in samples pre-acidified with metaphosphoric acid. Total vitamin C [dehydroascorbate (DHA)+AA] was determined by pre-treating acidified samples with the reductant 50 mM Tris(2-carboxylethyl)phosphine (TCEP) (Molecular Probes, Eugene, OR, USA) for 15 min prior to HPLC analysis. The DHA concentration was calculated by subtracting the measured ascorbate from the total vitamin C concentration. Full details of these methods are outlined in the online supplement.

Cell pellets were thawed on ice in 1 ml 100 mM sodium phosphate buffer (pH7.5), after which 150 µl zirconium breaker beads (0.7 mm, Stratech Scientific Limited, Newmarket Suffolk, UK) were added and the samples vortexed for 5 min. The resultant homogenate was then aliquoted for subsequent analyses. Cellular GSH was measured as outlined previously (20). Superoxide dismutase (SOD, kit number 706002), catalase (kit number 707002), and GPx (kit number 703102) activities were measured on cell supernatants using kits from the Cayman Chemical Company (Ann Arbor, Michigan, USA). Full details of these assays are summarised in the online supplement. Glutathione reductase activity was measured using an adapted version of the GSSG-reductase-DTNB recycling method, as outlined in the supplement. Total protein was employed as the denominator for all of the measurements made in the cell supernatants, as determined using the bicinchoninic acid method.

Metal-handling proteins were measured in untreated lavage using commercially available enzyme-linked immunosorbent assay (ELISA)-kits. Human transferrin and ferritin (undiluted) were quantified in BAL fluid samples using ELISA-kits from Alpha Diagnostic International, Inc., San Antonio, TX, USA. Human lactoferrin was quantified by ELISA supplied by OXIS Research International, Inc., Portland, OR, USA. All the analyses of antioxidant concentrations and activities were performed at the MRC-PHE Centre for Environment and Health, King's College London, London, UK.

### Statistics

Data were non-parametric, as assessed by the Shapiro–Wilk normality test, and were described using medians, with inter-quartile ranges. Comparison of airway inflammatory cells, antioxidant concentrations, and activities between the different groups were performed using the Kruskal–Wallis one-way analysis of variance by ranks, with post-hoc testing using the Mann–Whitney U test. Comparisons of individual groups were restricted to 1) healthy never smokers versus smokers with normal lung function; 2) smokers with normal lung function versus COPD current smokers; 3) healthy never smokers versus COPD ex-smokers; and 4) COPD ex-smokers versus COPD current smokers. In the comparisons 1) and 4), the effect of current smoking was addressed in non-COPD versus COPD groups, whereas in the comparisons 2) and 3), the effect of COPD was addressed when controlled for smoking history. Correlations between the measured endpoints were performed using the Spearman Rank Order Correlation. A *p*-value of <0.05 was considered significant.

## Results

### Airway inflammation

Sufficient recoveries, that is, with adequate volume to determine total airway and differential cells counts from BW and BAL were obtained from 9 of 11 COPD smokers, 15 of 16 COPD ex-smokers, 16 of 16 smokers with normal lung function, and 13 of 13 healthy never smokers. BAL-recovery was significantly lower (36%) in the COPD ex-smoking group relative to the healthy never smokers (50%) (Table 1). There was a significant increase in BAL macrophage numbers in smokers with normal lung function compared with healthy never smokers, as well as in COPD smokers compared with COPD ex-smokers (Table 2). In BAL, there was an increase in neutrophils in smokers with normal lung function compared with healthy never smokers (Table 2). Increases in BAL mast cell numbers were observed in the smokers with normal lung function relative to the healthy non-smokers, with a similar increase also apparent in the more proximal BW sample (Table 2). Equivalent increases were not observed in the COPD smokers when compared with COPD ex-smokers. Data on lymphocyte subsets have been published previously (21, 22).

**Table 2:** Differential white blood cell counts in BW and BAL fluids from healthy never smokers, smokers with normal lung function (LF), COPD ex-smokers, and COPD current smokers

	Healthy never smokers (*n*=13, 13^≠^, 13^§^)	Smokers with normal LF (*n*=16, 16^≠^, 16^§^)	COPD ex-smokers (*n*=16, 15^≠^, 15^§^)	COPD smokers (*n*=11, 8^≠^, 9^§^)	KW test
Bronchial wash					
Total cells	7.0 (5.6–11.2)	9.7 (7.9–19.9)	3.8 (2.1–10.2)	9.7 (3.4–14.5)	NS
Macrophages	6.2 (3.4–9.3)	8.7 (6.5–17.0)	2.0 (1.6–6.6)*c*	8.9 (3.2–13.0)	*P*=0.031
Neutrophils	1.2 (0.3–1.4)	0.4 (0.2–0.7)	0.6 (0.2–1.4)	0.5 (0.2–0.6)	NS
Lymphocytes	0.6 (0.3–0.8)	0.7 (0.4–0.9)	0.3 (0.1–0.5)*c*	0.2 (0.1–0.4)*b*	*P*=0.016
Eosinophils	0.0 (0.0–0.1)	0.1 (0.0–0.1)	0.0 (0.0–0.1)	0.0 (0.0–0.2)	NS
Mast cells	0.00 (0.00–0.01)	0.03 (0.01–0.04)*a*	0.00 (0.00–0.01)	0.00 (0.00–0.01)	*P*=0.027
Bronchoalveolar lavage fluid					
Total cells	17.1 (11.1–26.9)	39.7 (34.0–48.3)*a*	17.7 (14.7–23.8)	29.7 (27.4–45.6)*d*	*P*<0.001
Macrophages	14.1 (9.8–16.6)	36.6 (30.7–45.5)*a*	14.0 (11.3–20.7)	29.0 (26.1–39.3)*d*	*P*<0.001
Neutrophils	0.1 (0.1–0.2)	0.5 (0.3–0.8)*a*	0.3 (0.1–0.4)	0.1 (0.1–0.6)	NS
Lymphocytes	1.7 (0.9–3.9)	2.3 (1.5–3.2)	2.3 (1.1–3.2)	1.3 (0.6–1.5)	NS
Eosinophils	0.0 (0.0–0.1)	0.1 (0.0–0.1)	0.1 (0.0–0.2)	0.2 (0.1–0.3)	NS
Mast cells	0.01 (0.01–0.02)	0.06 (0.03–0.10)*a*	0.01 (0.00–0.02)	0.03 (0.03–0.07)	*P*=0.013

Cell numbers are presented as cell/mL*10^4^. All data are expressed as medians with either the inter-quartile or full range, as indicated. Significant differences across groups were assumed at the 5% level using the Kruskal–Wallis one-way analysis of variance by ranks (significance illustrated in the far right hand column), with post-hoc testing between specified groups performed using the Mann–Whitney U Test.

Comparisons of individual groups were restricted to smokers with normal lung function (LF) versus healthy never smokers (*a*, *P*<0.05), smokers with normal LF versus COPD current smokers (*b*, *P*<0.05), healthy never smokers versus COPD ex-smokers (*c*, *P*<0.05), and COPD current smokers versus COPD ex-smokers (*d*, *P*<0.05). Subject numbers given in parenthesis represent the subjects recruited, plus the number of subjects from which BW (≠) or BAL (§) was recovered for differential cell counts.

### Low molecular weight antioxidants

**Fig. 1:**
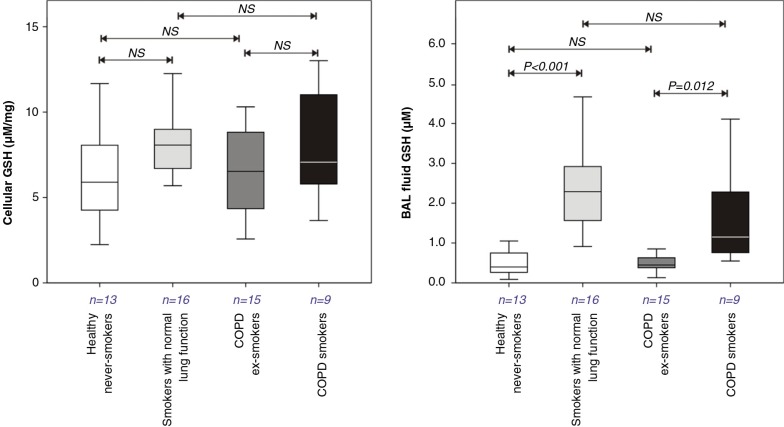
Glutathione concentration in BAL cells and bronchoalveolar lavage fluids recovered from healthy never smokers, smokers with normal lung function, COPD ex-smokers, and COPD smokers. Data are illustrated as box plots, with comparisons between groups performed using the Kruskal–Wallis test with post-hoc testing using the Mann–Whitney U test. *P*-values for Mann–Whitney U tests are given in the figure.

Due to limited BW recoveries from COPD patients, analyses were restricted to the more distal BAL fluid samples. Increased GSH concentrations were observed in both smokers with normal lung function (5.1-fold increase) and the COPD smokers (2.3-fold increase) relative to their non-smoking controls. This was not associated with a corresponding increase in intra-cellular GSH in cells obtained from BAL fluid (Fig. 1). Concentrations of BAL fluid GSSG were highly variable and not significantly increased with current smoking in smokers with normal lung function, 0.19 (0.05–0.42) µM, versus healthy never smoking controls, 0.06 (0.00–0.19) µM, or in COPD current smokers, 0.23 (0.13–0.47) µM versus COPD ex-smokers, 0.00 (0.00–0.71) µM. Similarly, cellular GSSG concentrations (µM) did not differ between the groups: smokers with normal lung function, 0.32 (0.13–0.45), versus healthy never smokers, 0.38 (0.21–0.50); COPD smokers, 0.22 (0.11–0.46) versus COPD ex-smokers, 0.31 (0.21–0.38).

**Fig. 2:**
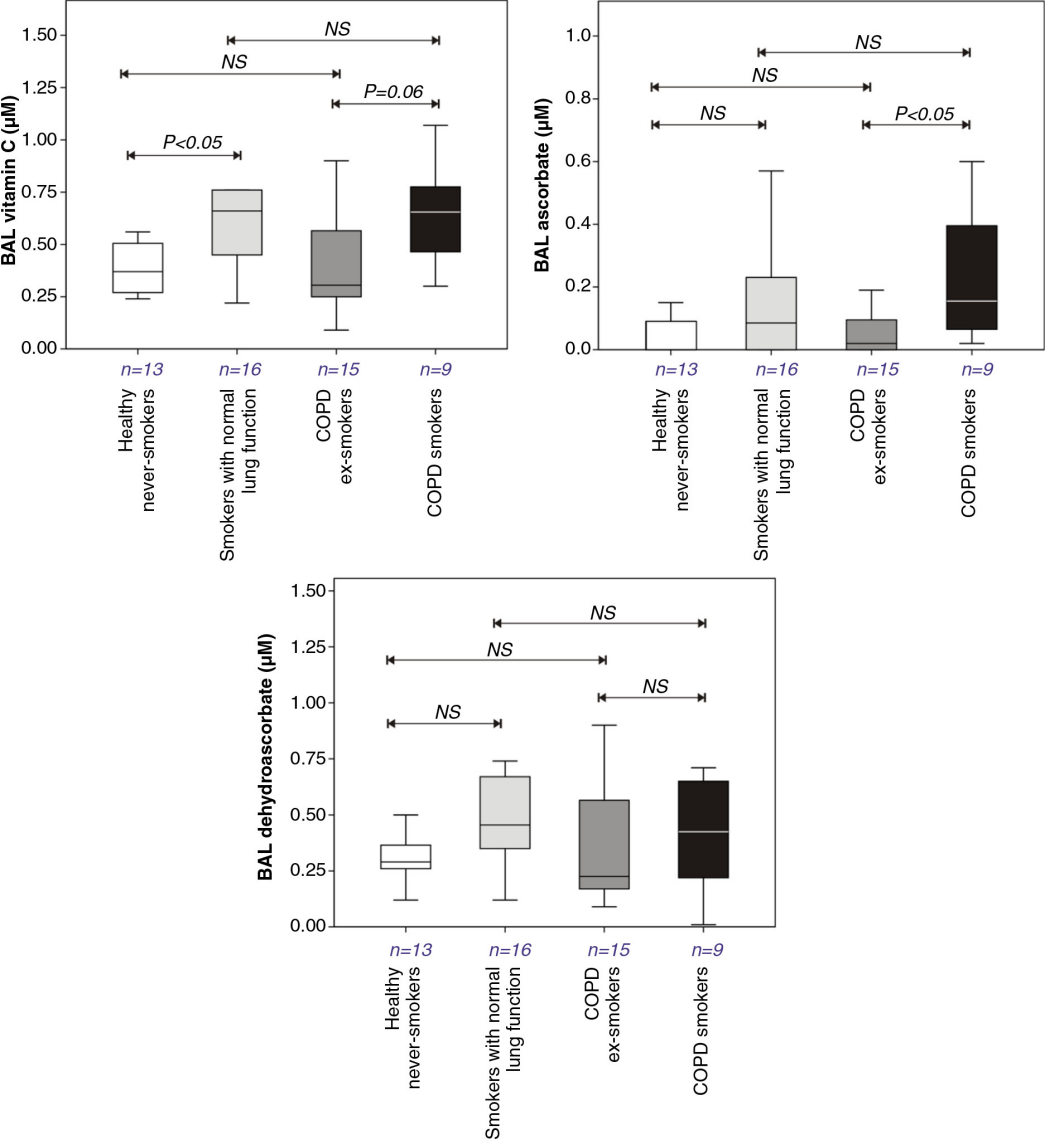
Total Vitamin C, ascorbate, and dehydroascorbate concentrations in bronchoalveolar lavage fluids recovered from healthy never smokers, smokers with normal lung function, COPD ex-smokers, and COPD smokers. Details of boxplots and statistical analysis are as described in Fig. 1.

Vitamin C concentrations were significantly augmented in BAL fluid in smokers with normal lung function, compared with healthy non-smoking controls (1.8-fold – Fig. 2). An increase (2.1-fold) was also observed in the COPD smoking group compared with the COPD ex-smokers, but this failed to attain statistical significance. These increases were only related to the subjects’ smoking status and not the presence of COPD. In the COPD patients, the significant increase in BAL fluid vitamin C concentrations with smoking reflected an increase in ascorbate (Fig. 2), though it was notable that ascorbate itself contributed only to a small fraction of the measured vitamin C pool and was often not measureable in the ex- or never smoking subjects. No evidence of altered BAL fluid urate concentrations was observed related to either smoking status or COPD diagnosis: 0.48 (0.28–0.70) µM – healthy never smoking controls, versus 0.41 (0.30–0.87) µM – smokers with normal lung function; 0.54 (0.35–0.75) µM – COPD ex-smokers, versus 0.66 (0.36–0.83) µM – COPD smokers.

### Chelator proteins

**Fig. 3:**
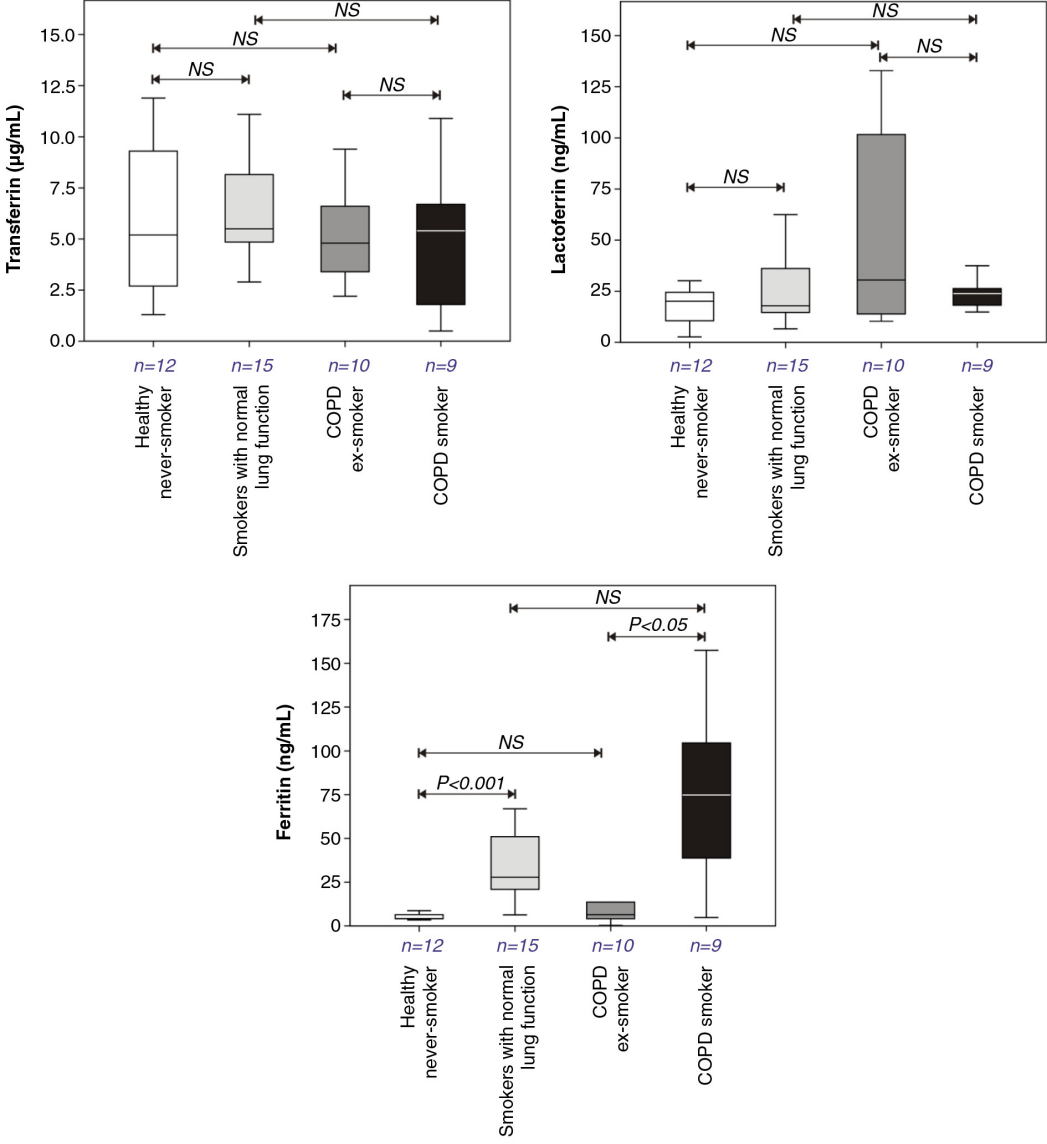
Transferrin, lactoferrin, and ferritin concentrations in bronchoalveolar lavage fluids recovered from healthy never smokers, smokers with normal lung function, COPD ex-smokers, and COPD smokers. Details of boxplots and statistical analysis are as described in Fig. 1.

A marked smoking-related increase in BAL fluid ferritin concentrations was observed in both smokers with normal lung function compared with healthy never smokers and in COPD smokers relative to COPD ex-smokers (Fig. 3). This response appeared to parallel the increased BAL fluid concentrations of glutathione and vitamin C reported in the smoking groups, though the concentrations were not quantitatively related. In contrast, lactoferrin and transferrin concentrations did not differ between the groups.

### Antioxidant enzyme activities

**Fig. 4:**
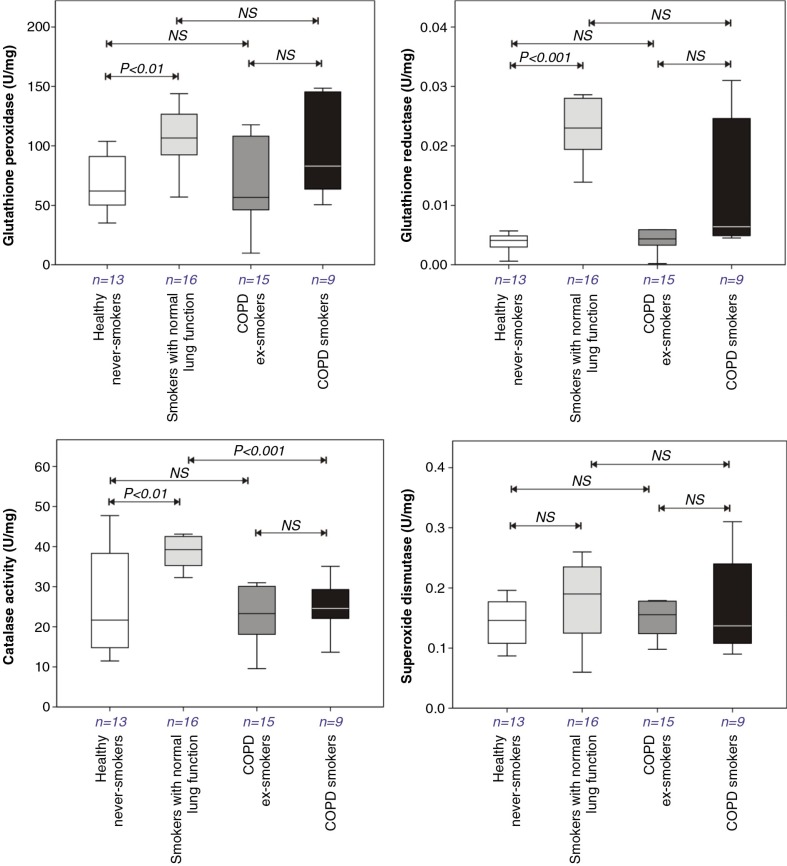
Antioxidant enzyme activities in BAL leukocytes recovered from the lungs of healthy never smokers, smokers with normal lung function, COPD ex-smokers, and COPD smokers. Details of boxplots and statistical analysis are as described in Fig. 1.

Significant increases in GPx [62.1 (49.8–93.6) versus 106.6 (91.2–137.7) U/g protein, *P*<0.01], GSSG-reductase [0.004 (0.003–0.005) versus 0.022 (0.019–0.028) U/g protein, *P*<0.001], and catalase activities [21.65 (13.65–40.48) versus 39.30 (35.30–43.10) U/g protein, *P*<0.01] were seen in BAL leukocytes from the smokers with normal lung function relative to the healthy never smokers (Fig. 4). Similar significant increases were absent in the smoking COPD patients. SOD activity was not altered with either disease or smoking status (Fig. 4).

### Correlation analyses

Correlation analyses were restricted to low molecular weight antioxidant concentrations and antioxidant enzyme activities that had been shown to differ between the patient groups and selected clinical endpoints related to impaired pulmonary function (FEV_1_ and reversibility), gas exchange (DL_CO_^SB^), and inflammation (BAL macrophage and neutrophil numbers). Overall, no coherent pattern of associations was seen (data summarised in Supplementary Tables e1–e4). While there was evidence of an association between BAL GSH and predicted FEV_1_ (Spearman's rho (ρ)=0.77, *P*=0.001) and predicted FVC (*ρ*=0.70, *P*=0.004) in the COPD ex-smokers, similar associations were not apparent in the COPD current smokers or when all COPD subjects (*n*=28) were considered (data not shown).

## Discussion

We examined evidence for impaired antioxidant defences in the peripheral airways of current and ex-smoking COPD patients compared with smokers with normal lung function and healthy never smokers. We found an increase in RTLF glutathione, vitamin C, and ferritin concentrations that were associated with smoking in both the COPD patients and in smokers with normal lung function. In smokers with normal lung function, current smoking was associated with increased GPx, glutathione reductase, and catalase activities in BAL leukocytes, whereas such adaptive increases were absent in the COPD-smoker group. RTLF and cellular antioxidant concentrations/activities were not simplistically related to smoking history, predicted FEV_1_, or the extent of chronic airway inflammation in this clinically stable group of COPD patients.

Evidence of increased airway oxidative stress in smokers and patients with COPD is largely based on the measurement of ROS, or markers of oxidative damage in EBC, due to the difficulty in obtaining airway lavage samples. Smokers and patients with COPD have been shown to have increased exhaled H_2_O_2_ compared with non-smokers (7, 23), with concentrations further elevated during exacerbations (24). Similarly, the concentrations of lipid oxidation products such as 8-iso-prostaglandin F2α (8-isoprostane) and thiobarbituric acid reactive substances (TBARS) have been shown to be elevated in EBC in healthy smokers relative to non-smokers, and markedly increased in patients with COPD (7, 8). Increased urinary concentrations of 8-iso-prostaglandin have also been shown in COPD, consistent with the presence of systemic oxidative stress (25). Oxidative damage to airway epithelium, endothelium, and neutrophils has been evaluated in lung tissue specimens obtained during lung resections from patients with and without COPD. In these specimens, increased staining for 4-hydroxy-2-nonenal (4-HNE)-modified proteins was observed in COPD patients (13), providing direct evidence of increased oxidative stress in the airways, with some evidence of a relation to lung function.

Our observation of increased RTLF GSH in smokers accords with previous observations (26). However, it still remains unclear whether this is a transient or sustained increase reflecting a regulated export mechanism; hence, adaptive or a passive release from dying cells. Consistent with our observations, a previous study in chronic smokers demonstrated that the extra-cellular increase in GSH occurred in the absence of increased cellular GSH concentrations and was in fact associated with a decreased expression of γ-glutamyl cysteine synthetase (γ-GCS) (27). This suggests that the increased extra-cellular GSH is unlikely to reflect a protective adaptation against chronic cigarette smoke exposure. These *in vivo* results are somewhat at odds with the reported induction of γ-GCS activity and γ-GCS heavy subunit mRNA in cultured immortalised human type II alveolar epithelial cells (A549) treated with cigarette smoke condensate (28). Increased RTLF GSH concentrations have previously been reported in a small group of COPD patients, though this may simply have reflected their smoking status, with concentrations shown to fall significantly during exacerbations (29).

There is currently an absence of studies examining RTLF ascorbate concentrations in COPD patients, with only a limited literature demonstrating cigarette smoke–induced decreases in lavage ascorbate in rats, as well as in smokers versus non-smokers (30). In the present study, we found significant increases in vitamin C in the smokers with normal lung function and in the smoking COPD patients, relative to their non-smoking controls, with ascorbate also increased in the smoking COPD patients. Similar to GSH, this response was unlikely to reflect a regulated airway response to cigarette smoke, as a significant proportion of the vitamin C pool was present as DHA, indicative of on-going oxidation in the lung. Urate concentrations were also determined, but concentrations did not differ between the groups, consistent with previous observations (14).

We also considered the concentrations of transferrin, lactoferrin, and ferritin in the lavages returns. These data demonstrated an increase in ferritin concentrations in currently smoking individuals (both in subjects with normal lung function and in COPD patients) relative to their non-smoking controls. This response appeared to parallel the increased concentrations of glutathione and vitamin C reported in the smoking groups, though there was no simple quantitative relationship. As ferritin is predominately an intra-cellular Fe storage protein; one explanation for this finding is that ferritin is being released into the RTLF as a function of cell death, with an associated release of intra-cellular antioxidants. This possibly suggests that the response is not a regulated adaptation, as discussed previously, but actually indicative of cell injury. Iron has been shown to be mainly deposited in ferritin within macrophages in COPD patients; thus, the current results are in line with release from dying macrophages (31). A similar increase in BAL fluid ferritin has previously been reported in human volunteers following instillation of iron-containing particles (32) though, in this later study, this was associated with parallel increases in lavage lactoferrin and decreased transferrin concentrations. There are also other potential explanations for the increase in RTLF ferritin concentrations, either reflecting the leakage of plasma soluble ferritin onto the surface of the lung or active secretion, as has been demonstrated in murine macrophages (33). The former suggestion seems unlikely in the absence of similar increases in abundant plasma antioxidants (urate) and proteins (transferrin), and the later secretion pathway has yet to be firmly established in humans.

Much of the current knowledge on cellular antioxidant status in COPD is based on the assessment of antioxidant enzyme activities in peripheral blood leukocytes (16, 34), and these studies have yielded ambiguous results. No measurements of antioxidant enzyme activities in airway cells obtained from COPD patients have been published. However, SOD activity in proximal airway lavage fluid has been observed to be similar between stable COPD patients and healthy controls (14). Red blood cell (RBC) SOD activity has been reported to be stable in COPD patients relative to non-smoking controls, despite increased plasma concentrations of lipid (malondialdehyde) and protein (carbonyls) oxidation markers (34). In contrast, during COPD exacerbations, increased RBC SOD activities were observed, while the activities of glutathione disulphide reductase and GPx fell (35). Erythrocytic GPx1 activity has been shown to be significantly decreased between GOLD Stages II, III, and IV in COPD patients relative to healthy controls, with the residual activity inversely related to systemic indices of inflammation (36). The observation of decreased GPx1 activity in peripheral blood has been confirmed in a study of 109 stable COPD patients and 51 controls, though notably with no incremental difference between patients at different disease stages (37). This study also demonstrated increased glutathione reductase activities in the peripheral blood from these subjects, parallel to the reduction in GPx1. In the present study, we failed to see equivalent antioxidant enzyme activity changes in airway leukocytes harvested from the lungs of COPD patients. We did, however, observe significant increases in the activities of GPx, glutathione reductase, and catalase in smokers with normal lung function, a response that was absent in the currently smoking COPD patients. SOD activity did not differ with COPD diagnosis or the presence of smoking.

Studies have demonstrated impaired nuclear factor (erythroid-derived 2)-like 2 (Nrf2) transcriptional activity in patients with emphysema, with reduced Nrf2 in lung tissue, and alveolar macrophages compared with age-matched controls (15). This occurred in parallel with increased Keap1 and Bach1 (a cap‘n'collar type of basic region leucine zipper factor that inhibits the expression of MAF proteins) protein expression, associated with decreased haeme oxygenase-1, NADPH quinone oxidoreductase, and GPx-2 mRNA. These data were interpreted as highlighting the increased susceptibility of the COPD airway to oxidative stress, through the loss of adaptive antioxidant and xenobiotic responses via impaired Nrf2 signalling. Our results are consistent with this view, as the expression of GPx, glutathione reductase, and catalase are up-regulated by Nrf2 inducers. Whilst we did not demonstrate any basal differences in antioxidant enzyme activities between the ex-smoking COPD and the healthy never smoking control groups, it is likely that such a functional impairment in Nrf2 signalling is only manifest when the airway is stressed, such as would occur as a result of cigarette smoke inhalation, or during exacerbations. In this study, all COPD patients were clinically stable, with no overt airway inflammation compared with the non-COPD controls. It is, therefore, unlikely that basal differences in antioxidant enzyme expression and activity would have been detected. These data, therefore, imply that the COPD airway has a decreased ability to respond to increased oxidative injury.

The current study sought to clarify RTLF and antioxidant defences in the peripheral lung of patients with COPD, but it should be acknowledged that a comprehensive consideration of the overall antioxidant network within the lung would require an appreciation of the concentration and activities of antioxidants within the epithelium of peripheral conducting airways and alveoli. This was not possible in the present study. Nevertheless, the results do extend the current knowledge of antioxidant defences in COPD, and these data represent the first report on antioxidant enzyme activities in alveolar leukocytes from this patient group. The non-induction of antioxidant enzymes in actively smoking COPD patients relative to age-matched smokers with normal lung function should be viewed in the context that only nine subjects were available in this group and clearly this finding requires future replication. The present study did not explore the basis for decreased antioxidant enzyme activity in COPD smokers and so we cannot at this point state whether this reflects decreased protein expression or post-translational oxidative inactivation. We also observed substantial bronchodilator reversibility within the ex-smoking COPD group. No specific atopy test was included, but the COPD patients had a smoking history of at least 10 pack-years without any obvious record of asthma or allergy. Thus, we have no clear explanation to this observation.

## Conclusions

The current study demonstrates that cigarette smoking induces the up-regulation of several antioxidants present at the air–liquid interface; low molecular weight sacrificial antioxidants in the RTLF and antioxidant enzyme in airway leukocytes, in both subjects with normal lung function and in COPD patients. Importantly, the present data are consistent with a blunted adaptive intra-cellular antioxidant enzyme response to cigarette smoke in airway leukocytes in individuals with COPD. As a consequence, in patients with COPD, airway leukocytes are likely more susceptible to oxidative injury and the induction of redox-sensitive signalling pathways upon exposure to cigarette smoke or inflammatory, cell-derived oxidants. This inability to up-regulate antioxidant defences to mitigate against oxidative stress would, therefore, contribute to the vicious cycle of inflammation, oxidative stress and tissue destruction/remodelling seen in COPD, contributing to the symptomatic deterioration in these patients.

## Supplementary Material

Cigarette smoke–induced induction of antioxidant enzyme activities in airway leukocytes is absent in active smokers with COPD
