# Supplementary material for: Cigarette smoke–induced induction of antioxidant enzyme activities in airway leukocytes is absent in active smokers with COPD
Source: Eur Clin Respir J. 2015 Jul 16;2:10.3402/ecrj.v2.27837. doi: 10.3402/ecrj.v2.27837 (PMC4629722; doi:10.3402/ecrj.v2.27837)
Supplement: Cigarette smoke–induced induction of antioxidant enzyme activities in airway leukocytes is absent in active smokers with COPD [file ECRJ-2-27837-s001.pdf]

SUPPLEMENTARY MATERIAL FOR THE PAPER

Expanded methodology

Collection and preparation of bronchoalveolar lavage samples

Bronchoscopy was performed on an outpatient basis, following an overnight fast, using a flexible video bronchoscope (Olympus BF IT160, Tokyo, Japan) inserted through the mouth with the subject in the supine position. Pre-medication with atropine (1 mg) was given subcutaneously 30 minutes prior to bronchoscopy to reduce airway mucous secretion, with Lidocain (4% and 2%) sprayed onto the airways to achieve topical anaesthesia. Bronchial wash (BW) was performed by infusing two aliquots of 20 mL sterile sodium chloride (NaCl), pH 7.3 at 37°C into the lingular or middle lobe, which was gently sucked back after each infusion. The recovered aspirates were kept as separate aliquots on wet ice. Bronchoalveolar lavage (BAL) was performed immediately after these small volume infusions by the instillation and immediate aspiration of 3 consecutive aliquots of 60 mL saline. The recovered aspirates were pooled and placed on wet iced prior to transport to the laboratory processing.

The chilled bronchial wash (BW) and bronchoalveolar lavage (BAL) fluids were filtered through a nylon filter (pore diameter 100µM) to remove the mucus and centrifuged (400 g, 15 minutes, at 4°C). After centrifugation, the cell pellet was separated from the supernatant and resuspended in PBS. The total number of cells was counted and adjusted to a final concentration of 10^6 cells/ml. Differential cell counts were carried out on cyto-centrifuge preparations stained with May-Grűnwald Giemsa, with a 400 total cell count per slide. Further description of these

methods is available (S1). The resultant supernatant was treated as follows: an aliquot of lavage fluid (450 μL) was treated with 50 μL of 50% metaphosphoric acid (MPA), vortexed for 30 seconds and centrifuged at 13,000 rpm for 5 minutes (4ºC) to remove protein. The resultant supernatant was then stored at −80ºC within 30 minutes of collection until required for analysis. Samples for GSH and GSSG determination were treated with the metal chelator desferoxamine mesylate and the synthetic antioxidant butylated hydroxytoluene both at 2 mM, 5 µl of each to 490 µl of lavage, prior to storage at -80°. The remaining lavage was untreated, but immediately aliquoted and stored at -80°C.

Antioxidant and oxidative damage marker analyses

In general, due to limited BW recoveries from COPD patients, all antioxidant and antioxidant enzyme analyses were restricted to the more distal BAL fluid samples.

Ascorbate and urate: Ascorbate (AA) and urate (UA) were measured simultaneously by reverse phase HPLC with electrochemical detection (S2). Pre-acidified and deproteinated samples stored at -80ºC in 5% MPA were thawed on wet ice. Lipid extraction was achieved by transferring 400 µl aliquots of these acidified samples to eppendorfs containing 50 µl of 5% MPA and 200 µl of heptane chilled to 4ºC, followed by vortexing for 60 seconds and centrifugation at 13,000g for 5 minutes (4ºC). The resultant lower layer was then carefully decanted into amber HPLC vials for analysis. All sample processing was performed on wet ice with care taken to protect the samples from light at all times. A Gilson 234 auto-sampler was used to inject 20 µl aliquots of each sample for analysis on a 5 µm C18 column (4.6 x 150 mm) from Phenomenex, eluted with a 0.2 mM K_2HPO_4-H_3PO_4 mobile phase containing 0.25 mM octanesulphonic acid (pH 2.1) at a flow-

rate of 1.5 ml/min. An E&EG amperometric electrochemical detector was used for detection with the voltage set at 400 mV and a current sensitivity of 0.2 µA. Ascorbate and urate concentrations were determined against appropriate standards (AA range: 0-12.5 µM; UA: 0-25 µM). Total vitamin C (dehydroascorbate (DHA) + ascorbate) was measured by pre-treating the 400 µl aliquots of acidified sample with 50 µl 50 mM Tris(2-carboxylethyl)phosphine (TCEP) in 5% MPA (Molecular Probes, Eugene, Oregon USA) for 15 minutes and then performing the lipid extraction and HPLC analysis as described above. The DHA concentration was then calculated by subtracting the measured ascorbate concentration from the total vitamin C concentration.

Glutathione determinations: Reduced Glutathione (GSH) concentrations were calculated by measuring total glutathione (GSx) and glutathione disulphide (GSSG) concentrations, using the GSSG-reductase-DTNB recycling method developed by Tietze (S3) and modified by Baker (S4). For total GSH, 50 µl aliquots of cell lysate were added to 96-well ELISA microplate wells and 100 µl of DTNB reaction mix added to achieve final concentrations of 0.15 mM DTNB, 0.2 mM NADPH and 1 U glutathione reductase (from Baker's yeast) in 100 mM sodium phosphate buffer (pH 7.5) in each well. The plate was then transferred to a plate reader and the immediate rate of change in absorbance at 405nm followed for two minutes at 30°C, with absorbance measurements made every 10 seconds, and with mixing between measurements. Total GSH concentrations were measured by comparison against a set of GSSG standards (0-6.6 µM). GSSG concentrations were measured by pre-treating samples with 2-vinyl pyridine for 1 hour and then analysing as outlined for total GSH. GSSG concentrations were also measured by

comparison with a set of GSSG standards (0-3.3 μM). The reduced GSH concentration was then calculated by subtracting double the GSSG concentration from the total GSH concentration.

Antioxidant enzyme analysis: All antioxidant enzyme activities were measured using commercially available kits. These assays are described below with a brief description of the principle of each assay.

Superoxide dismutase: Total superoxide dismutase (SOD) activity was measured in cell lysates using an assay kit from the Cayman Chemical Company following manufacturer's instructions. Briefly, cell lysates were diluted 5-fold with sample buffer (50 mM Tris-HCl, pH 8.0). 10 μl of each diluted sample were then incubated for 20 minutes in a reaction mixture containing the assay buffer (50 mM Tris-HCl, pH 8.0; 0.1 mM diethylenetriaminepentaacetic acid (DTPA); 0.1 mM hypoxanthine), a tetrazolium salt radical detector and xanthine oxidase, in a 96-well plate. The absorbance was read at 450nm and a linearised rate (Abs_{450}[lowest standard]/Abs_{450}[sample]) calculated for each sample relative to a set of SOD standards (0-0.25 U/ml).

Catalase: Catalase activity was measured using an assay from the Cayman Chemical Company following manufacturer's instructions. Cell lysates were initially diluted 10-fold with sample buffer (25 mM potassium phosphate, pH 7.5, containing 1 mM EDTA and 0.1% BSA). Subsequently 20 μl of the diluted sample was pipetted into a 96-well plate and 100 μl assay

buffer (100 mM potassium phosphate, pH 7.0) and 30 µl methanol added to each well. The reaction was initiated by the addition of 20 µl 0.04 M H_2O_2. After 20 minutes incubation the reaction was terminated by the addition of 30µl 10 M potassium hydroxide. 30 µl of the chromagen 4-amino-3-hydrasino-5-mercapto-1,2,4-triazole (Purpald) was then added and incubated for 10 minutes. Finally, 10 µl potassium periodate was incubated in the wells for 5 minutes and the absorbance read at 540nm. Catalase activity was quantified by comparison against a set of formaldehyde standards (0-75 µM) prepared in sample buffer. One unit for catalase was defined as the amount of enzyme that will cause the formation of 1.0 nmol of formaldehyde per minute at 25°C.

Glutathione peroxidase: Glutathione peroxidase (GPX) activity was measured indirectly, using an assay kit from the Cayman Chemical Company, based on a coupled reaction with Glutathione reductase (GSR). Reduction of H_2O_2 by GPX produces GSSG, which is then recycled to GSH by GSR and NADPH. The oxidation of NADPH to NAPH+ causes a decrease in absorbance at 340nm that is proportional to the activity of GPx. The assay was carried out following manufacturer's instructions. Briefly, 20 µl of each undiluted cell lysate were added to 100 µl assay buffer (50 nM Tris-HCl, pH 7.6; 5 mM EDTA) and 50 µl of a pre-mixed co-substrate mixture (containing NADPH, GSH and GSR) in a 96-well plate. The reaction was initiated by addition of 20 µl cumene hydroperoxide and the absorbance at 340nm read every minute for 6 minutes, with mixing between measurements. GPx activity was then calculated by dividing the rate of change in absorbance by the extinction coefficient of NADPH (0.00373 μM^{-1}). One unit of GPx was defined as the amount of enzyme that will cause the oxidation of 1.0 nmol of NADPH to NADP+ per minute at 25°C.

Glutathione reductase: Glutathione reductase (GSR) was measured using an adapted version of the GSSG-reductase-DTNB recycling method. 50 µl lysate were added to plate wells and 100 µl of DTNB reaction mix added to give final concentrations of 0.15 mM DTNB, 0.2 mM NADPH and 0.6 mM GSSG. The immediate rate of change of absorbance at 405nm was followed for 2 minutes. Activity was quantified by comparison against a range of GSR standards (0-0.6 U/ml) prepared in a sodium phosphate buffer containing BSA (100 mM anhydrous NaH_2PO_4; 1 mM EDTA; 1 mg/ml BSA, pH 7.5)

Additional results

Correlation analyses: Correlation analyses were performed between those antioxidant and oxidative damage marker concentrations which had been shown to differ between the patient groups and selected clinical endpoints related to impaired pulmonary function (FEV_1 and reversibility), gas exchange ($TL_{CO}SB$) and inflammation (BAL macrophage and neutrophil numbers). The results of these analyses are summarised in supplementary Tables s1 – s4 for healthy never-smokers, healthy smokers, COPD ex-smokers and COPD current smokers. Overall these analyses failed to show any coherent pattern of associations related to smoking status or the severity of COPD. Whilst there was evidence of a statistically significant association between BAL GSH and predicted FEV_1 (Spearmans rho (ρ)=0.77, P=0.001) and airway reversibility following bronchodilation with 1mg of terbutalin (ρ=0.70, P=0.004) in the COPD ex-smokers (Table s3), similar associations were not apparent in the COPD current smokers (Table s4) or when all COPD subjects (n=28) were considered in the analysis (data not shown).

e-Table 1: Correlation (Spearman Rank Order Correlations) matrix for healthy never-smokers.

		**Clinical Parameters**					**BAL cells**		**BAL fluid antioxidants**			**BAL cell antioxidants**		
		Pack years	FEV_1 % predicted	FVC_1 % predicted	TL_{CO}^{SB}	Reversibility	Macrophages	Mast cells	GSH	Vitamin C	Ferritin	Glutathione peroxidase	GSSG reducatse	Catalase
Age	*Correlation Coefficient*	.	-.336	-.562	.039	.039	-.420	.077	.188	-.133	.417	-.095	-.328	.376
	Sig. (2-tailed)	.	.262	.046	.900	.900	.153	.802	.539	.696	.156	.769	.298	.229
Pack years	*Correlation Coefficient*		.	.	.	.	.	.	.	.	.	.	.	.
	Sig. (2-tailed)		.	.	.	.	.	.	.	.	.	.	.	.
FEV_1 % predicted	*Correlation Coefficient*			.512	-.233	-.233	.486	.057	-.326	-.137	-.330	.471	.549	**-.595**
	Sig. (2-tailed)			.074	.444	.444	.092	.853	.277	.687	.271	.122	.064	**.041**
FVC_1 % predicted	*Correlation Coefficient*				-.039	-.039	.321	.057	-.069	-.136	-.368	.258	.516	-.223
	Sig. (2-tailed)				.900	.900	.285	.852	.822	.690	.216	.419	.086	.487
TL_{CO}^{SB}	*Correlation Coefficient*						.	.	.	.	.	.	.	.
	Sig. (2-tailed)						.	.	.	.	.	.	.	.
Reversibility	*Correlation Coefficient*						-.154	-.080	.154	.100	.466	.480	.481	.219
	Sig. (2-tailed)						.615	.795	.615	.769	.108	.114	.113	.495
Macrophages	*Correlation Coefficient*							.225	.170	**.697**	.083	-.084	.042	-.403
	Sig. (2-tailed)							.460	.578	**.017**	.788	.795	.897	.194
Mast Cells	*Correlation Coefficient*								.390	.120	-.232	-.321	-.172	-.501
	Sig. (2-tailed)								.188	.725	.445	.308	.593	.097
GSH	*Correlation Coefficient*									**.633**	.382	-.112	-.070	.263
	Sig. (2-tailed)									**.036**	.198	.729	.829	.409
Vitamin C	*Correlation Coefficient*										.508	-.132	-.160	.119
	Sig. (2-tailed)										.111	.699	.639	.728
Ferritin	*Correlation Coefficient*											.168	.121	**.587**
	Sig. (2-tailed)											.601	.707	**.045**
Glutathione peroxidase	*Correlation Coefficient*												**.841**	.140
	Sig. (2-tailed)												**.001**	.664
GSSG reductase	*Correlation Coefficient*													-.074
	Sig. (2-tailed)													.820

Significant associations (Spearman rho and *P*-value, n=11-13) are highlighted in bold text.

e-Table 2: Correlation (Spearman Rank Order Correlations) matrix for smokers with normal lung function.

		Clinical Parameters					BAL cells		BAL fluid antioxidants			BAL cell antioxidants		
		Pack years	FEV_1 % predicted	FVC_1 % predicted	TL_{CO}^{SB}	Reversibility	Macrophages	Mast cells	GSH	Vitamin C	Ferritin	Glutathione peroxidase	GSSG reducatse	Catalase
Age	*Correlation Coefficient*	.382	-.394	-.289	-.387	.167	-.003	-.166	.163	.327	-.031	-.185	**-.699**	.181
	Sig. (2-tailed)	.145	.131	.278	.138	.536	.991	.538	.547	.254	.909	.509	**.005**	.518
Pack years	*Correlation Coefficient*		-.257	-.404	.151	.487	.477	.273	.165	.177	.269	-.384	-.436	.223
	Sig. (2-tailed)		.336	.121	.578	.055	.062	.306	.540	.544	.314	.157	.119	.425
FEV_1 % *predicted*	*Correlation Coefficient*			**.814**	.384	.310	-.272	-.023	.121	.411	-.154	.456	**.678**	.110
	Sig. (2-tailed)			**.000**	.142	.242	.309	.932	.655	.144	.568	.088	**.008**	.698
FVC_1 % *predicted*	*Correlation Coefficient*				.131	.230	-.334	-.345	-.010	.349	-.161	.465	**.706**	.088
	Sig. (2-tailed)				.628	.392	.206	.191	.970	.222	.550	.081	**.005**	.756
TL_{CO}^{SB}	*Correlation Coefficient*					**.553**	.285	.333	.141	.046	.444	.093	.317	-.064
	Sig. (2-tailed)					**.026**	.284	.208	.602	.875	.085	.742	.270	.820
Reversibility	*Correlation Coefficient*						.374	.327	.456	**.542**	.322	-.032	-.031	.079
	Sig. (2-tailed)						.154	.217	.076	**.045**	.223	.909	.917	.781
Macrophages	*Correlation Coefficient*							.426	.324	.306	**.584**	**-.707**	-.356	-.150
	Sig. (2-tailed)							.100	.222	.287	**.017**	**.003**	.211	.594
Mast Cells	*Correlation Coefficient*								.097	.012	.230	-.420	**-.535**	-.169
	Sig. (2-tailed)								.722	.967	.392	.119	**.048**	.547
GSH	*Correlation Coefficient*									**.883**	.415	.175	-.015	.132
	Sig. (2-tailed)									**.000**	.110	.533	.958	.639
Vitamin C	*Correlation Coefficient*										.405	.029	.026	.062
	Sig. (2-tailed)										.151	.923	.928	.834
Ferritin	*Correlation Coefficient*											-.245	.104	-.004
	Sig. (2-tailed)											.379	.725	.990
Glutathione peroxidase	*Correlation Coefficient*												**.759**	.207
	Sig. (2-tailed)												**.002**	.459
GSSG reductase	*Correlation Coefficient*													-.020
	Sig. (2-tailed)													.946

Significant associations (Spearman rho and *P*-value, n=14-16) are highlighted with bold text

e-Table 3: Correlation (Spearman Rank Order Correlations) matrix for COPD ex-smokers.

		**Clinical Parameters**					**BAL cells**		**BAL fluid antioxidants**			**BAL cell antioxidants**		
		Pack years	FEV_1 % predicted	FVC_1 % predicted	TL_{CO}^{SB}	Reversibility	Macrophages	Mast cells	GSH	Vitamin C	Ferritin	Glutathione peroxidase	GSSG reducatse	Catalase
Age	*Correlation Coefficient*	-.117	-.048	.091	.101	-.025	-.322	.019	.326	-.305	-.491	.311	.023	.403
	Sig. (2-tailed)	.655	.856	.727	.700	.925	.224	.944	.236	.336	.063	.352	.947	.219
Pack years	*Correlation Coefficient*		**.492**	**.631**	.406	.185	.295	.440	.387	.264	-.120	.160	-.027	-.205
	Sig. (2-tailed)		**.045**	**.007**	.106	.476	.268	.088	.154	.408	.670	.639	.936	.544
FEV_1 % predicted	*Correlation Coefficient*			**.569**	**.601**	-.083	.338	.055	**.771**	.455	-.052	**.635**	**.690**	.133
	Sig. (2-tailed)			**.017**	**.011**	.752	.200	.841	**.001**	.137	.854	**.036**	**.019**	.696
FVC_1 % predicted	*Correlation Coefficient*				**.546**	.232	.250	.197	**.700**	.414	-.303	.418	.136	.145
	Sig. (2-tailed)				**.023**	.369	.351	.464	**.004**	.181	.273	.201	.689	.670
TL_{CO}^{SB}	*Correlation Coefficient*					.110	.474	.475	**.687**	.084	-.204	.573	.427	-.100
	Sig. (2-tailed)					.673	.064	.063	**.005**	.795	.467	.066	.190	.770
Reversibility	*Correlation Coefficient*						-.029	-.002	.116	.095	.325	.055	.100	-.509
	Sig. (2-tailed)						.914	.996	.680	.770	.237	.873	.770	.110
Macrophages	*Correlation Coefficient*							.213	.036	-.270	**.579**	.455	.536	-.482
	Sig. (2-tailed)							.428	.899	.397	**.024**	.160	.089	.133
Mast Cells	*Correlation Coefficient*								.195	-.262	-.173	-.294	-.359	**-.644**
	Sig. (2-tailed)								.486	.410	.538	.381	.278	**.033**
GSH	*Correlation Coefficient*									**.691**	-.267	.574	.565	.077
	Sig. (2-tailed)									**.013**	.337	.065	.070	.821
Vitamin C	*Correlation Coefficient*										-.102	-.042	.176	.201
	Sig. (2-tailed)										.753	.915	.651	.604
Ferritin	*Correlation Coefficient*											.227	.555	**-.609**
	Sig. (2-tailed)											.502	.077	**.047**
Glutathione peroxidase	*Correlation Coefficient*												**.791**	.318
	Sig. (2-tailed)												**.004**	.340
GSSG reductase	*Correlation Coefficient*													.091
	Sig. (2-tailed)													.790

Significant associations (Spearman rho and *P*-value, n=11-17) are highlighted with bold text

e-Table 4: Correlation (Spearman Rank Order Correlations) matrix for COPD smokers.

		**Clinical Parameters**					**BAL cells**		**BAL fluid antioxidants**			**BAL cell antioxidants**		
		Pack years	FEV_1 % predicted	FVC_1 % predicted	TL_{CO}^{SB}	Reversibility	Macrophages	Mast cells	GSH	Vitamin C	Ferritin	Glutathione peroxidase	GSSG reducatse	Catalase
Age	*Correlation Coefficient*	.000	-.296	.009	.036	.300	-.633	.271	.167	-.238	-.250	-.143	-.333	-.143
	Sig. (2-tailed)	1.000	.377	.979	.915	.370	.067	.480	.668	.570	.516	.736	.420	.736
Pack years	*Correlation Coefficient*		-.164	-.301	-.409	.500	.150	**.712**	.500	.000	.233	**.714**	.643	.524
	Sig. (2-tailed)		.630	.368	.212	.117	.700	**.031**	.170	1.000	.546	**.047**	.086	.183
FEV_1 % predicted	*Correlation Coefficient*			**.794**	.041	**-.729**	.350	-.034	-.233	.190	-.200	-.381	-.381	-.405
	Sig. (2-tailed)			**.004**	.905	**.011**	.356	.931	.546	.651	.606	.352	.352	.320
FVC_1 % predicted	*Correlation Coefficient*				-.224	**-.658**	.209	-.068	-.335	.000	-.360	-.371	-.419	-.419
	Sig. (2-tailed)				.508	**.028**	.589	.862	.379	1.000	.342	.365	.301	.301
TL_{CO}^{SB}	*Correlation Coefficient*					.082	-.250	-.254	-.450	.071	.383	-.690	-.571	**-.714**
	Sig. (2-tailed)					.811	.516	.509	.224	.867	.308	.058	.139	**.047**
Reversibility	*Correlation Coefficient*						-.533	.492	.083	-.119	.133	.667	.571	.310
	Sig. (2-tailed)						.139	.179	.831	.779	.732	.071	.139	.456
Macrophages	*Correlation Coefficient*							-.186	.233	.238	.483	-.048	.143	.238
	Sig. (2-tailed)							.631	.546	.570	.187	.911	.736	.570
Mast Cells	*Correlation Coefficient*								.610	.366	.034	.317	.146	-.024
	Sig. (2-tailed)								.081	.373	.931	.444	.729	.954
GSH	*Correlation Coefficient*									.524	.167	.333	.286	.405
	Sig. (2-tailed)									.183	.668	.420	.493	.320
Vitamin C	*Correlation Coefficient*										.476	-.071	.036	-.036
	Sig. (2-tailed)										.233	.879	.939	.939
Ferritin	*Correlation Coefficient*											-.024	.190	-.024
	Sig. (2-tailed)											.955	.651	.955
Glutathione peroxidase	*Correlation Coefficient*												**.952**	**.857**
	Sig. (2-tailed)												**.000**	**.007**
GSSG reductase	*Correlation Coefficient*													**.881**
	Sig. (2-tailed)													**.004**

Significant associations (Spearman rho and *P*-value, n=8-11) are highlighted with bold text.

Supplement references

S1 Mudway IS, Stenfors N, Blomberg A, Helleday R, Dunster C, Marklund SL, Frew AJ, Sandström T, Kelly FJ. Differences in basal airway antioxidant concentrations are not predictive of individual responsiveness to ozone: a comparison of healthy and mild asthmatic subjects. Free Radic Biol Med. 2001;31(8):962-74.

S2 Iriyama K, Yoshiura M, Iwamoto T, Ozaki Y. Simultaneous determination of uric and ascorbic acids in human serum by reversed-phase high-performance liquid chromatography with electrochemical detection. Anal Biochem. 1984;141(1):238-43.

S3 Tietze F. Enzymic method for quantitative determination of nanogram amounts of total and oxidized glutathione: applications to mammalian blood and other tissues. Anal Biochem. 1969;27(3):502-22

S4 Baker MA, Cerniglia GJ, and Zaman A. Microtiter plate assay for the measurement of glutathione and glutathione disulphide in large numbers of biological samples. Anal Biochem. 1990;190(2):360-65
